# Insect Meals and Insect Antimicrobial Peptides as an Alternative for Antibiotics and Growth Promoters in Livestock Production

**DOI:** 10.3390/pathogens12060854

**Published:** 2023-06-20

**Authors:** Ewelina Patyra, Krzysztof Kwiatek

**Affiliations:** Department of Hygiene of Animal Feedingstuffs, National Veterinary Research Institute, Partyzantów 57 Avenue, 24-100 Puławy, Poland; kkwiatek@piwet.pulawy.pl

**Keywords:** antimicrobial peptides (AMPs), insects, glycine-rich peptides, defensins, moricins, cecropins, proline-rich peptides, mode of action, livestock health, Commission Regulation (EU) 2017/893

## Abstract

The extensive use of antibiotics in animal production has led to the development of antibiotic-resistant microorganisms and the search for alternative antimicrobial agents in animal production. One such compound may be antimicrobial peptides (AMPs), which are characterized by, among others, a wide range of biocidal activity. According to scientific data, insects produce the largest number of antimicrobial peptides, and the changing EU legislation has allowed processed animal protein derived from insects to be used in feed for farm animals, which, in addition to a protein supplement, may prove to be an alternative to antibiotics and antibiotic growth promoters due to their documented beneficial impact on livestock health. In animals that were fed feeds with the addition of insect meals, changes in their intestinal microbiota, strengthened immunity, and increased antibacterial activity were confirmed to be positive effects obtained thanks to the insect diet. This paper reviews the literature on sources of antibacterial peptides and the mechanism of action of these compounds, with particular emphasis on insect antibacterial peptides and their potential impact on animal health, and legal regulations related to the use of insect meals in animal nutrition.

## 1. Introduction

The discovery of antibiotics in the first half of the 20th century and their introduction to the treatment of infectious diseases was one of the greatest historical achievements of medicine, and it was the basis for the development of methods of saving lives and protecting health [1]. Antibiotics began to be used in breeding animals in the 1950s. They primarily had to prevent diseases causing the greatest losses in animal husbandry, improve the use of nutrients in feed, and lower the cost of feeding [2].

Currently, one of the main problems in modern medicine is that there is a daily fight against pathogenic microorganisms resistant to available antibiotics. The widespread use of antibiotic therapy has significantly reduced the effectiveness of this group of drugs, leading to difficulties in controlling the increasing number of infections. The spread of antibiotic-resistant pathogenic microorganisms seriously threatens animal and human health and causes economic losses [3,4]. Threats related mainly to the spread of drug resistance among pathogenic microorganisms have led to attempts to limit the use of antibiotics worldwide in veterinary medicine, agriculture, and the feed industry [5].

Maintaining the production efficiency, health, and welfare of farm animals while limiting the use of antibiotics in their breeding requires the search for other substances that will be an alternative to antibiotics [5]. Nowadays, more and more interest is aroused by the huge and extremely diverse group of peptides showing direct antimicrobial activity.

Antimicrobial peptides (AMPs) have been described as an evolutionarily ancient weapon against microbial infections. Antimicrobial peptides are metabolites synthesized by both prokaryotic and eukaryotic organisms, which are part of the body’s non-specific defense and play a significant role in innate immunity. As part of the innate immune system, AMPs provide immediate effective non-specific defense against infections [6]. Antimicrobial peptides exhibit activity against a wide range of Gram-positive bacteria, Gram-negative bacteria, fungi, and viruses. In addition, some of them can inhibit pro-inflammatory reactions, accelerate wound healing, and neutralize bacterial toxins and biofilm formation processes [7,8,9].

Since the first antimicrobial peptide was isolated from the skin of the African clawed frog *Xenopus laevis*, which was named magainins, much attention has been devoted to AMPs as compounds that may constitute a new class of antibiotics. AMPs are a component with innate immunity against pathogenic organisms and have evolved in most living organisms for more than 2.6 billion years [10,11]. Due to their natural origin, they may turn out to be less toxic than the antibiotics used so far. In addition, the changing nutritional conditions of animals and the search for full-value sources of protein in animal nutrition have led in recent years to the legalization of the use of insect meals in feed as a partial replacement of, e.g., soybean. In addition, there are documented indications that antimicrobial peptides synthesized by insects may have a beneficial effect on the health of animals that are fed feeds with the addition of insect protein.

For this reason, this article aims to characterize insect antimicrobial peptides and present the latest literature data on their therapeutic potential in the context of the growing problem of antibiotic resistance as well as their possible application in livestock nutrition as an alternative to antibiotics.

## 2. Antimicrobial Peptides

In the course of evolutionary development, animals and plants developed several defense mechanisms—an important element of this defense is antimicrobial peptides. They are isolated from almost all organisms, both plant and animal, where they are an important part of the immune system. Antimicrobial peptides are present on all surfaces exposed to the external environment. They are probably one of the most basic and, at the same time, the most primitive defense mechanisms. Common features of antimicrobial peptides are low molecular weight (compared to other peptides), linear or cyclic structure, and cationic structure or hydrophobic nature [12]. As a rule, they are monogenically coded and constitute a characteristic feature of a given species. Information on the number of described AMPs is available in the DRAMP database [Data Repository of Antimicrobial Peptides (DRAMP)] [13]. Currently, 3791 AMPs from six kingdoms have been reported in this database, including 2519 from animals, 824 from plants, 431 from bacteria, 6 from fungi, 4 from archaea, and 7 from protozoa [14,15]. The first reports of AMP appeared in the 1980s; at that time, cecropin was isolated from the moth pupa and two magainin peptides were isolated from frog skin.

Naturally occurring AMPs can vary in size and structure, but most of these substances contain 12 to 50 amino acids per molecule. These are mainly positively charged peptides (from +2 to +9) with the dominant share of cationic lysine and arginine residues, and about 50% with hydrophobic residues [16,17]. Recently, a few anionic AMPs have been reported that have a net charge ranging from −1 to −7, and a length from 5 to circa 70 amino acid residues [18]. These properties mean that these substances can bind to the negatively charged target cell surface. In Gram-positive bacteria, peptides interact with peptidoglycan and teichoic acid, thereby displacing divalent ions from the cell while allowing them to bind to negatively charged lipids outside the cell membrane. In the case of Gram-negative bacteria, AMPs interact with lipopolysaccharide, also displacing divalent ions that stabilize it, while the anti-tumor activity of AMPs results from the binding of tumor cells with negatively charged membrane lipids [19]. In general, AMPs show a wide range of antibacterial, anticancer, antifungal, and antiviral activity [20,21].

AMPs have been divided into four groups based on their structure: β-sheet, α-helical, cyclic, and elongated. Antimicrobial peptides may consist of a single sheet or helix, but may also have a more complex structure. Elongated peptides have no recognizable structure motives. However, they contain large amounts of specific amino acids such as tryptophan, arginine, histidine, and glycine [20].

## 3. Source of Antimicrobial Peptides

AMPs are produced by bacteria (bacteriocins, lantibiotics), fungi, plants (e.g., thionine in wheat), invertebrates, and vertebrates. The task of AMPs, regardless of their origin, is the body’s protective function. The highest known number of antimicrobial peptides is produced by vertebrates (69%). AMPs are part of the immunity of living organisms. Many of the compounds are produced by bacterial strains. In the case of microorganisms, AMPs are used to eliminate other cells from the environment on a competitive basis, e.g., in conditions of insufficient nutrients. A group of antimicrobial peptides produced by bacteria is bacteriocins. They are distinguished by a narrow spectrum of activity compared to other AMPs, covering only single strains. It has been proven that strains of Gram-negative bacteria are usually resistant to the action of bacteriocins produced by Gram-positive strains [22].

Another group of antimicrobial peptides is compounds produced by plant organisms. Plants produce AMPs in response to infections caused by bacteria, viruses, or fungi, and, in addition to their antimicrobial activity, they can act as antioxidants or inhibit certain enzymes. Most peptides isolated from plants belong to compounds rich in cysteine residues. Examples of AMPs produced by plant organisms are thionines, snakins, and defensins [23]. The ɣ-thionines, which belong to defensins, are isolated from barley and wheat and are active against bacteria and fungi as well as *Leishmania donovani*, giving rise to hope in combating parasitic invasions [24].

Among the antimicrobial peptides, the largest group is compounds produced in animal organisms. Their presence has been confirmed in many cells, including blood components (macrophages, lymphocytes, neutrophils), mucosal epithelial cells, or body fluids (e.g., tear fluid). AMPs produced by animals exhibit a broad spectrum of activity and high antimicrobial activity [16]. Cathelicidins belong to one of the best-described groups of antimicrobial peptides. There are, among others, organisms from mammals, fish, and amphibians. The most famous representative of cathelicidines is the LL-37 peptide. Its function is to regulate the inflammatory response, promote wound healing, and act as an antimicrobial agent. In mammals, AMP is produced, inter alia, in neutrophils, macrophages, and epithelial cells. In the human body, AMPs are mainly produced by cells in the tissues of the skin, airways, lungs, or intestines. The group of human antimicrobial peptides includes mainly defensins and cathelicidines (cationic peptides, often rich in cysteine residues) and histatines (peptides present mainly in saliva, rich in histidine residues) [6]. Literature reports mention the deficiency of defensins and cathelicidines as one of the main factors in the development of atopic dermatitis and Crohn’s disease [25].

Insect antimicrobial peptides are synthesized in hemocytes and the adipose body (the functional equivalent of the liver in mammals) and secreted into the hemolymph, where they participate in the systemic response against pathogens. Peptides are also synthesized in epithelial cells that participate in local reactions including the site of the microbial intrusion or the point where the continuity of the insect’s body layer is broken [26]. Some peptides are also expressed in the middle gut [27,28]. In invertebrates, peptide synthesis may be constitutive, but it may also be induced by injury or infection of the organism [29].

## 4. Insect Antimicrobial Peptides

Insects are the organisms from which the greatest amount of peptides are isolated. A single insect produces a mixture of 15–20 peptides, the concentration of which in the hemolymph increases rapidly during infection. Their presence in the hemolymph enables the body’s systemic response to infection, while peptides synthesized in epithelial cells participate in local reactions involving the gates of infection [30].

With over a million described species, insects make up the largest class of organisms in the world. Insects show adaptability to repeated changes and resistance to a wide range of pathogens [31]. The mechanism of resistance developed by insects is associated with an immune system based solely on the innate immune response, which allows for a quick and broad response to attacking organisms [32,33]. In recent years, insect AMPs have been increasingly used in pharmacy as well as in agriculture. With a growing number of identified peptides that can inhibit human pathogens, insect AMPs are of great interest for biomedical applications. Insect AMPs represent a highly promising alternative to overcome medical problems associated with antibiotic resistance.

The first insect AMP, the cecropin, was identified in 1980 from the pupae of *Hyalophora cecropia* [34,35]. It is believed that a given species of insects has a characteristic set of immune peptides that differ in their biochemical properties and mechanism of action about microorganisms, which enables effective control of a wide range of microorganisms (Gram-positive and Gram-negative bacteria, fungi, and protozoa). AMPs have been characterized by representatives of various orders of insects: Diptera, Coleoptera, Hymenoptera, Hemiptera, Lepidoptera, Trichoptera and Odonata. In insects, AMPs are divided into three most important groups: linear α-helical cecropins; defensins; peptides rich in glycine; and proline residues. Insects are considered an excellent source of antimicrobial peptides because of their huge biodiversity.

### 4.1. Defensins (Cysteine-Rich Peptides)

The largest group of insect antimicrobial peptides are defensins. Defensins are also found in a variety of invertebrates, vertebrates, and plants. In insects, defensins constitute a very large group of antimicrobial peptides. They were described, inter alia, with representatives of Diptera, Hemipter, Coleoptera, Lepidoptera, Odonata and Hymenoptera. Currently, almost 170 defensins are known to be present in invertebrates. In insects, defensin genes are mainly expressed in the fat body of insects, but in hematophages (blood-sucking insects), e.g., *Anopheles gambiae* or *Stomoxys calcitrans*, also in the middle intestine. The first insect defensins: sapecin and defensins A and B were obtained from the *Sarcophaga peregrina* (Diptera) cell line and from the hemolymph of immunized *Phormia terranovae* (Diptera) larvae, respectively [36]. Insect defensins contain 32–52 amino acid residues (3–6 kDa) [37]. The number of amino acids forming the α-helix and the two β-sheets is highly conserved in all known insect defensins, while the length of the N-terminal loop is variable. In evolutionarily older insects such as dragonflies (Aeshna), the defensin loop is shorter compared to the Coleoptera or Hemiptera defensins [38,39]. The defensins A (3.8 kDa) and B (3.84 kDa) *Chironomus plumosus* (Diptera) have a spatial structure similar to *Protophormia terranovae* defensins, but their N-terminal loops are four amino acid residues shorter, which is interesting because such short loops characterize defensins of species evolutionarily distant from Diptera (dragonflies, scorpions, clams). In representatives of the same order of insects, the sequences of defensins show a high degree of homology, while between different orders of insects, this phenomenon is not observed. The mechanism of action of defensins is to change the permeability of the cytoplasmic membrane of Gram-positive bacteria, creating channels in it. Against Gram-negative bacteria, defensins usually lack antibacterial activity [40,41]. The mechanism of action of defensin A isolated from consists in the formation of oligomers in the bacterial membrane, which leads to its damage, partial depolarization, loss of K+ ions from the cell, inhibition of respiratory processes, and reduction in the cytoplasmic level of adenosine triphosphate (ATP) [42]. Insect defensins are especially active against Gram-positive bacteria such as *Bacillus subtilis*, *Staphylococcus aureus*, *Bacillus megaterium*, and *Micrococcus luteus*. However, some of the insect defensins also show antimicrobial activity against Gram-negative bacteria such as *E. coli* [43]. Insects, in addition to the production of antimicrobial defensins, are able to synthesize cysteine-rich peptides that have antifungal activity. To date, three antifungal defensins have been characterized: [43] heliomicin from the tobacco budworm *Heliothis virescens*; drosomycin from *Drosophila melanogaster*; and termicin from the termite *Pseudacanthotermes spiniger* [44].

### 4.2. Cecropins (α-Helical AMPs)

Cecropins are the best-studied group of peptides. The first described insect antimicrobial peptide was cecropin. It was isolated in 1980 from the hemolymph of immunized *Hyalophora cecropia pupae*. So far, cecropins have been described in insects belonging to various orders, including Diptera, Coleoptera, and Lepidoptera.

The group of cecropins includes a large number of antibacterial and toxic peptides isolated from various lepidopteran and dipteran species. Cecropins are small proteins with about 35 amino acid residues. In insects, the most common are cecropins A, B, and D, which consist of 35–37 residues without cysteine. Insect cecropins are active against both Gram-positive and Gram-negative bacteria [45]. Cecropins can lyse bacterial cellular membranes, inhibit proline uptake, and cause leaky membranes [46,47]. Insect cecropins also have other names including lepidopteran, bactericidin, sarcotoxin, etc. [48]. To date, several insect cecropins have been studied both structurally and biologically, evaluating their in vitro activity. Cecropin A has been shown to have a stabilized α-helical structure thanks to which it reduces both NADP+ and glutathione levels, causing oxidative stress by creating reactive oxygen species, but the mechanism of action is not known so far [49]. Cecropin A shows activity against the fungus *Beauveria bassiana* in silkworm larvae [50]. Cecropin B, which is a linear cationic peptide, shows the highest antibacterial activity among the entire cecropin family. The publications contain information that cecropin B reduces the bacterial load of *E. coli*, and the concentration of endotoxins in plasma shows antifungal activity against *Candida albicans* [51]. Some cecropins have anti-inflammatory activity [27]. Some cecropins and cecropin derivatives (SB-37 and Shiva) have also been shown to be active against parasites, including Trypanosome and Plasmodium [52,53,54]. In addition, they may inhibit cancer cell proliferation and HIV-1 replication [55]. Cecropins have no hemolytic activity, but ponericins (particles similar to cecropin from ant venom) are lethal to erythrocytes and are potent insecticides. Cecropins, in addition to antibacterial activity, may affect the development of Chagas disease, viruses, and parasites that cause malaria [56].

### 4.3. Moricins

Moricins, described only in Lepidoptera, were first isolated from the hemolymph of immunized *Bombyx mori* caterpillars. Unlike cecropins, the moricin molecule forms a single α-helix structure, the amphipathic amino terminus of which the hydrophobic carboxyl terminus is essential for antimicrobial activity. The structure on which this polypeptide acts is the bacterial cell membrane [57]. Moricins show bactericidal activity, especially against Gram-positive bacteria: *B. cereus*, *S. aureus*, and *Streptococcus pyogenes*, but also against Gram-negative *E. coli* [42,58]. Gram-positive bacteria are more sensitive to moricin than Gram-negative bacteria. The α-helical structure of the moricin molecule makes it similar to cecropins, except that moricin does not contain an amino nitrogen. In addition, there are no disulfide bridges in the moricin molecule. Moricin, an induced immune protein with a broad spectrum of antibacterial activity, is one of the main humoral factors of the antibacterial defense of the body cavity of the mulberry silkworm.

The antifungal activity of moricin is weak and basically targeted at yeast. Moricin, with a strong effect on Gram-positive bacteria, together with mulberry silkworm cecropins, which act mainly on Gram-negative bacteria, effectively eliminate infections of the body cavity of the *Bombyx mori* caterpillar [59].

### 4.4. Proline-Rich Peptides

Proline-rich AMPs are isolated from insects and mammals and exhibit antibacterial activity mainly against Gram-negative bacteria. Proline-rich insect antimicrobial peptides typically contain 20–35 amino acid residues and function by penetrating and crossing the bacterial cell membrane and entering the periplasmic space [60]. In a cell, peptides inhibit intracellular processes such as the transport system. Due to their special properties, they are among the potential cell-penetrating peptides capable of internalizing impermeable drugs into bacteria and eukaryotic cells [61]. Characteristics of proline-rich AMPs are drosocin and apidicin. Drosocin has been isolated from insects belonging to the order of insects Lepidoptera, Hymenoptera, Hemiptera, and Diptera. The mechanism of action of proline-rich antibacterial peptides is by interfering with DNA and RNA synthesis and by binding to nucleic acids. In addition, proline-rich peptides have been shown to have specific macromolecular targets. Proline-rich peptides exhibit antibacterial activity against *Pseudomonas aeruginosa*, *Escherichia coli*, *Acinetobacter baumannii*, and *Klebsiella pneumoniae*. Studies conducted for pyrhocoricin, apidaecin, and drosocin have shown that these antibacterial peptides may act in a different, precise way involving the molecular binding of the peptide to bacterial DNaK, thereby inhibiting the action of ATPase and preventing the folding of proteins supported by chaperones [39,62].

### 4.5. Glycine-Rich Peptides

The mechanism of action of glycine-rich peptides is the destruction of cell membranes. These peptides are active against Gram-negative bacteria as well as active against fungi and tumor cells. Glycine-rich peptides including sarcotoxin IIA, hymenoptaecin, attacin, diptericin, and coleoptericin have been identified in various insect species, including: *Glossina morsitans Westwood* (Diptera), *Bombyx mori* L. (Lepidoptera), *Heliothis virescens Fabricius* (Lepidoptera), *Musca domestica* L. (Diptera), *Samia ricinidaturidini*, (Lepidoptera: Noctuidae), and *Trichoplusia ni Hübner* (Lepidoptera). The high amount of glycine residues (14–22%) in AMPs affects the function of the tertiary structure of proteins by blocking the synthesis of outer membrane proteins in dividing Gram-negative bacteria such as *E. coli*, thus disrupting the integrity of the cell wall [63].

## 5. Mode of Action of AMPs

AMPs show a wide spectrum of antibacterial activity, they are active against Gram-positive and Gram-negative bacteria but also show activity against fungi and viruses. However, their mechanism of action differs from that of antibiotics. The mechanism of action of antibiotics is based on four main ways: (1) inhibition of cell wall synthesis, protein synthesis orucleic acid synthesis, and damage to cell membrane; (2) mutation of antibiotic function sites; (3) mutations in genes encoding DNA gyrase and Topoisomerase IV; or (4) reduction in antibiotic intercellular concentration [23]. As already mentioned, antibacterial peptides show activity against Gram-positive and Gram-negative bacteria, fungi, eukaryotic parasites, and viruses as well as against cancer cells. Among the mechanisms of action of antibacterial peptides, we distinguish interference in cell metabolism and targeting cytoplasmic components and disruption of the cell membrane. These mechanisms of action lead to cell death [64]. The first contact that occurs between the pathogen and the antibacterial peptide is an electrostatic or hydrophobic interaction, which depends on the lipid composition of the pathogen cell membrane [65].

Four models can be distinguished among the mechanisms of membrane activity of peptides. The first is the “barrel stave” model characteristic of peptides with an α-helix or β-sheet structure based on the creation of specific channels. Peptides bind to the cell membrane and insert themselves into the hydrophobic core of the membrane. This causes pores and leakage of cytoplasmic material and a reduction in the membrane potential. Thus, peptides damage cell membranes, disrupting the ionic homeostasis of cells and leading to the dysfunction and death of bacterial cells. In the “carpet” model, peptides accumulate on the surface of the lipid bilayer without penetrating it. The peptides interact electrostatically with the hydrophilic heads of phospholipids, covering the membrane surfaces like a carpet. At high concentrations of peptides, the membrane is destabilized and ruptured, and the cell’s membrane potential is lowered and the cytoplasm components leak. This is a similar mechanism to that of a detergent. In the toroidal pore model, AMPs align perpendicular to the lipid bilayer and induce a local curvature with pores partly formed by peptides and partly by phospholipid heads. The resulting transient lipid–peptide supramolecule is referred to as the “toroidal pore”. In contrast to the “barrel stave” model, pores are formed when the hydrophobic and hydrophilic system is disturbed. Moreover, in this model, sometimes AMPs molecules penetrate the cytoplasm, interacting directly with intracellular components. Peptides whose antimicrobial activity is related to the mechanism of “disordered ring pores” may be anchored to the membrane at different angles and not perpendicular to the membrane as in the “ring” mechanism [36].

**Figure 1:**
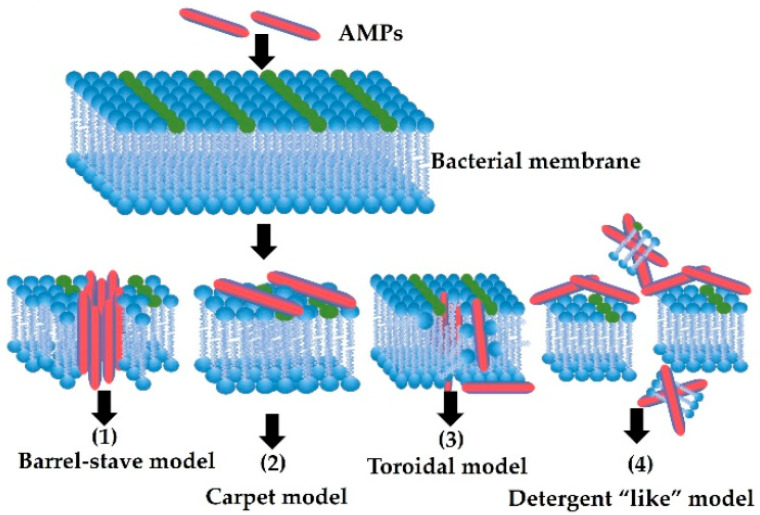
Mechanism of action of AMPs. Membrane-active AMPs interrupt the integrity of the membrane by forming different pores as in the following models: 1—barrel stave model; 2—carpet model; 3—toroidal pore model; 4—detergent-like model [23].

Despite very intensive research, some reports in the literature show that the described mechanisms of action of antimicrobial peptides do not fully explain the observed phenomena [66]. It has been noticed that many peptides have the ability to penetrate the cytoplasmic membrane and accumulate inside the cell. It has been proven that AMP peptides intercalate with DNA and cause disturbances in its structure. There is a change in the angle of the helix, the distance between the base pairs, and the diameter of the molecule DNA. This leads to an inhibition of efficient replication and transcription. The degree of damage to DNA as a genetic template depends on the structure of the AMP peptide [67]. The modes of action of AMPs are shown in Figure 1.

## 6. Mechanisms of Resistance to AMPs

In the course of evolutionary progress, microorganisms have developed a number of defense mechanisms against antimicrobial substances. Currently, the resistance of pathogenic microorganisms to the action of antibacterial substances is one of the biggest public health problems. Since the discovery of penicillin in the 1920s by Alexander Fleming and other antibiotics, they have been used on a large scale in both human and veterinary medicine. However, their excessive use has led to the development of microbial resistance and the lack of therapeutic effect [68,69]. The defense mechanisms of microorganisms against the action of antibiotics are different from the mechanisms of action of antibacterial peptides, which are associated with changes in the cell membrane, which affect the binding of peptides and/or their insertion and membrane permeability [70,71].

Microbes have the ability to develop defense mechanisms that allow them to avoid the attack of known, previously used substances. However, the immunization of microorganisms to AMP peptides is an extremely slow process. Evolutionary changes concern both the AMP peptides and the microbes themselves. Based on a comparison of the time of exposure of microorganisms to the action of AMP peptides (calculated practically from the beginning of the existence of AMP-producing organisms) with the time of exposure to antibiotics (calculated from the moment they were introduced into treatment) as well as the frequency of such interactions, it can be concluded that the resistance of microorganisms to AMP peptides is insignificant. However, after more than eighty years of use, many antibiotics are ineffective today. Due to the extremely slow, but progressing, process of microbial resistance to AMP peptides, the mechanisms leading to the limitation of binding and incorporation of the AMP peptide into the microbial cell membrane and changes in the permeability of the cytoplasmic membrane should be analyzed [72].

The modification of the outer cell membrane occurs as a result of the acylation of lipid A of lipopolysaccharide, described in the case of, for example, *Salmonella enterica* and *Staphylococcus aureus* [73]. The production of polysaccharide capsules is another mechanism of building resistance of microorganisms, especially important in the case of inducing resistance to defensins by *Klebsiella pneumoniae* [74]. Another possible way of acquiring resistance to AMP is the production of enzymes capable of degrading AMP peptides. Such enzymes are produced by bacteria from the Proteus, Salmonella, Pseudomonas, Enterococcus, and Streptococcus families [75]. Many pathogens have efflux pumps whose activity depends on the concentration of potassium ions, thanks to which it is possible to remove, for example, defensins from the bacterial cell, and at the same time the uptake of potassium ions inside it [76]. Insect AMPs represent a highly promising alternative to overcome medical problems associated with antibiotic resistance.

## 7. Effect of Antimicrobial Peptides on Livestock Health

Antimicrobial peptides offer great hope due to the global problem of increasing bacterial resistance to antibiotics. The mechanism of action of insect AMP has evolved over more than a century of evolution and is very conservative, suggesting a low risk of bacterial resistance. Additionally, AMP insecticidal insecticides can also protect the body against viruses and fungi [77].

Insect meals are rich in bioactive compounds and nutrients, so they can partially replace conventional sources of protein in animal nutrition, such as soybean meal and fishmeal. Insect meals, in addition to high protein content, are rich in short- and medium-chain fatty acids and chitin, as well as peptides and polysaccharides produced by insects that may have antibacterial and/or immunostimulating effects. Immunostimulation and enhanced disease resistance suggest that a small addition of insect meal to the diet can be a potent supplement to provide “healthy animal feed” or “natural non-specific oral vaccines” to stimulate the immune system of animals. They can be used as a “preventive treatment” against stressful situations for animals, such as transport or seasonal changes in temperature, which are usually associated with an increased risk of infection [77]. In addition, in the publication of Yun and Lee [51], they proved that AMP present in insect meals leads to the improved performance of animals, improved nutrient digestibility, and support of normal intestinal morphology and function.

Xiao et al. [78] showed that AMPs can protect piglets from challenges with mycotoxin deoxynivalenol (DON) present in the feed. The research showed that DON could enhance intestinal permeability, damage villi, cause epithelial cell apoptosis, and inhibit protein synthesis, whereas composite antimicrobial peptides improved intestinal morphology and promoted intestinal epithelial cell proliferation and protein synthesis, indicating that AMPs may repair the intestinal injury induced by DON. Moreover, AMP as an immune molecule can reduce inflammation, improve the composition of the intestinal microflora, and strengthen the intestinal barrier function of weaned piglets [78].

It has been shown that AMPs are probably responsible for the reduction in the bursa of Fabricius, which plays an important role in the differentiation of B lymphocytes in birds. The results of Józefiak et al. [79] showed that the inclusion of small amounts (0.05 to 0.2%) of full-fat insect meals in feed affected the gastrointestinal microbiome and lowered the pH value in the cecum of poultry. This acidification may indicate a bacteriostatic role of insect meals in the digestive tract of poultry and does not cause the development of bacterial resistance. Islam and Yang [80] showed that the addition of 0.4% full-fat mealworm (*Tenebrio molitor*) or wood eater (*Zophoba morio*) meal reduced mortality and increased IgG and IgA antibody levels in *Salmonella* and *E. coli* infected broiler chickens. The authors suggest that the chitin present in mealworm and woody grub meal had a probiotic effect that was able to act as a natural antibiotic. The addition of black soldier fly meal—*Hermetia illucens*—increases the amount of bacteria such as *Bacteroides-Prevotella*, *Clostridium cocoides-Eubacterium rectale*, and *Streptococcus* spp.*/Lactococcus* spp. in the cecum. *C. cocoides* is the commensal microbiota of the gastrointestinal tract and is considered a group of bacteria that play an important role in immunology, nutrient absorption, and disease processes. Free-range chickens fed *Tenebrio molitor* meal showed increased *Firmicutes* and decreased *Bacteroidetes phyla*, and a higher *Firmicutes*/*Bacteroidetes* ratio. The *Firmicutes* type plays an important role in feed digestion and host health, while higher *Firmicutes*/*Bacteroidetes* ratios are associated with a bacterial profile with a higher energy-harvesting capacity. Increased levels of commensal bacteria may have a beneficial effect on health by stimulating the immune system [79]. For pigs, Ji et al. [81] in in vivo studies on piglets suggested a positive effect of insect AMPs on reducing the incidence of diarrhea in weaned piglets fed feed with the addition of 5% *Tenebrio molitor*, *Musca domestica*, or *Zophobas morio* [80].

In an in vivo study conducted on weaned piglets from commercial farms, the AMP complex provides a mixture of lactoferrin, cecropin, defensin, and plectasin (2 g and 3 g kg^−1^ feed), improving growth performance, reducing the incidence of diarrhea, and increasing the survival rate of weaned piglets in comparison with untreated sows [77]. In vitro results suggest that compounds containing insect AMP complexes have tagging of individual peptides and small molecules of thibiotics. Accordingly, the use of insect meal providing a variety of different AMPs as an animal feed additive could open up new possibilities in animal production. An interesting solution in animal production may be dietary supplementation with an active substance, an antimicrobial fraction of proteins extracted from dried full-fat insect meal [5].

Antibacterial peptides have a beneficial effect on nutrient digestibility, intestinal morphology, and intestinal microflora, and thus on growth performance in animals. In addition, they can affect the activity of neutrophils, T lymphocytes, and dendritic cells. Unlike antibiotics, which can induce microbial resistance, the antimicrobial mechanisms of AMPs may provide a strategy to prevent the development of bacterial resistance [72]. In addition to direct antibacterial effects on microbes, antimicrobial peptides may provide protection through other mechanisms that contribute to maintaining normal intestinal homeostasis and modulating host inflammatory responses [6,82]. In pigs and broilers, the presence of antimicrobial peptides in the ration improves growth performance, positively changes the intestinal microflora, strengthens the immune function, and supports the digestibility of nutrients and intestinal health [83]. It is documented that early weaning in pig farms can increase the incidence of diarrhea and mortality and cause stress and growth delays in piglets [84]. The authors conducted an experiment in which 5% insect meals were added to the feed at the beginning of the weaning of piglets, and it was confirmed that the incidence of diarrhea significantly decreases between 15 and 28 days. The study by Ji et al. [81] showed that the use of *T. molitor*, *M. domestica*, and larvae as a source of protein reduces the occurrence of diarrhea in piglets. AMPs present in insect meals are thought to help prevent intestinal inflammation and damage to the intestinal mucosa. Antimicrobial peptides provide great hope because of the global build-up problem of bacterial resistance to antibiotics. Antimicrobial peptides from insects can also protect against viruses and fungi. In addition, unlike antibiotics, for example, AMPs derived from *Hermetia illucens* can provide a defensive effect that protects animals from infection by pathogenic microbes and alter the behavior of cells in response to external damage [85]. However, the biological effects of peptides depend on their bioavailability, which is determined by their resistance to gastrointestinal digestion [86]. Protease susceptibility can be overcome using various strategies, including D-isomerization, incorporation of chemical compounds, cyclization, and use of peptide mimetics [87]. However, to ensure the biological effects of peptides, it is important to carry out in vitro and in vivo studies that confirm their stability, absorption capacity, and mechanism of action, which increase and diversify their nutraceutical and pharmaceutical applications [88].

## 8. Legal Regulations Regarding the Use of Insects in Feeding Farm Animals

Studies have shown that insects are a sustainable source of animal feed in many countries around the world due to their ability to provide nutrients. Insects have a higher feed processing efficiency and use nutrients better than animals. In addition, insects produce fewer ammonia and greenhouse gas emissions and use less water than those produced by traditional livestock. Insect farming has important environmental aspects, such as reduced consumption of drinking water and feed, which can be byproducts of the agro-food industry to meet food and feed safety requirements. The current “feed ban” and the decrease in fish catches and the increased demand for feed for farm animals and aquaculture resulted in a sharp decrease in the availability of fishmeal and fish oil, with a simultaneous increase in the prices of these materials. For this reason, efforts are being made to search for new sources of proteins of animal origin. In 2017, Commission Regulation (EU) 2017/893 on 24 May 2017 was issued amending Annexes X, XIV, and XV to Commission Regulation No. 142/2011 with regard to the provisions on processed animal protein [89]. Pursuant to this amendment, processed animal protein derived from farmed insects and intended for the production of animal feed, obtained exclusively from the following insect species, was authorized for use as feed: black soldier fly (*Hermetia illucens*) and common housefly (*Musca domstica*), yellow mealworm (*Tenebrio molitor*), house cricket (*Acheta domesticus*), banded cricket (*Gryllodes sigillatus*), and field cricket (*Gryllus assimilis*). The available literature data indicate that processed insect protein may be an alternative and potentially important source of protein for use in animal nutrition. Processed insect protein is characterized by the presence of a readily available source of protein, lipids, carbohydrates, certain vitamins and minerals, and antimicrobial peptides. In addition to those mentioned, processed insect protein contains chitin, chitosan, and lauric acid, which have immunomodulatory and antibacterial properties too [61].

## 9. Conclusions

Due to the increasing number of microorganisms resistant to antibiotics and the implementation of antibiotic protection programs and attempts to limit their use in human and veterinary medicine, scientists and industry are forced to look for alternative substances. In addition, the growing demand for protein sources in the nutrition of farm animals made the European Union allow the use of insect meal in animal nutrition as a supplement of protein in animal feed. It has been documented that processed insect proteins have a positive effect on the intestinal microflora, improve immunity, and prevent bacterial infections in farm animals. Insect meals can be a good source of protein and antimicrobial peptides that can be used as alternatives to antibiotics in animal production, including supporting animal growth and health, treating infections, and preserving food. However, much remains to be achieved to facilitate the large-scale production of insect AMPs and find ways to use them effectively in livestock.

## Data Availability

Not applicable.
